# Smoke, Scars, and Survival: A Six-Year Analysis of Burn Mortality in a Resource-Limited Appalachian ICU

**DOI:** 10.7759/cureus.82199

**Published:** 2025-04-13

**Authors:** Armein Rahimpour, Eli McFeeley, Nathan Fox, Kassidy Price, Taylor Adkins, Curtis W Harrison, David Denning, Paul Bown, Rahman Barry

**Affiliations:** 1 General Surgery, Marshall University Joan C. Edwards School of Medicine, Huntington, USA; 2 Plastic and Reconstructive Surgery, Marshall University Joan C. Edwards School of Medicine, Huntington, USA

**Keywords:** burn care, burn injuries, burn intensive care unit, clinical outcomes, resource-limited settings

## Abstract

Background: Despite recent advances in burn management, burn injuries remain a major global cause of morbidity and mortality, with rural and underserved populations, such as those in Appalachia, being disproportionately affected. Contributing factors include limited access to specialized care and a high prevalence of comorbidities. Understanding the prognostic factors associated with mortality in adult burn patients is critical for guiding clinical care and resource allocation, particularly in resource-limited settings.

Methods: This retrospective study analyzed data from adult burn patients aged 18 to 65 admitted to the only Burn Intensive Care Unit (BICU) in West Virginia, located at Cabell Huntington Hospital, between January 2017 and January 2023. A total of 748 patients were included. Variables analyzed included demographics, comorbidities (diabetes mellitus, chronic obstructive pulmonary disease (COPD), smoking history, home oxygen use), injury characteristics (inhalation injury, total body surface area burned (TBSA)), and clinical outcomes (total hospital duration (THD), total ventilation duration (TVD)). Categorical and continuous variables were compared between survivors and non-survivors using chi-square and t-tests, respectively. Multivariate logistic regression was used to identify independent predictors of mortality.

Results: The cohort consisted of 748 patients with a mortality rate of 3.6% (n = 27). Non-survivors were significantly older (mean age 56.1 vs. 40.2 years, *p *< 0.001), had higher mean TBSA (28.3% vs. 6.3%, *p *< 0.001), longer hospital stays (15.3 vs. 8.9 days, *p* = 0.012), and longer ventilation durations (18.5 vs. 6.7 days, *p* < 0.001). Inhalation injury was present in 66.7% of deceased patients compared to 14.1% of survivors (*p* < 0.001). Comorbidities such as diabetes (51.9% vs. 12.5%, *p* < 0.001), COPD (63.0% vs. 12.3%, *p *< 0.001), and home oxygen use (55.6% vs. 8.2%, *p *< 0.001) were significantly more prevalent in non-survivors. Smoking was also significantly associated with mortality (81.5% vs. 45.9%, *p *= 0.001). In the logistic regression analysis, independent predictors of mortality included TBSA (OR 1.15 per 1% increase, 95% CI: 1.10-1.21, *p* < 0.001), TVD (OR 1.08 per day, 95% CI: 1.02-1.14, *p *= 0.009), smoking history (OR 2.34, 95% CI: 1.15-4.78, *p *= 0.018), and inhalation injury (OR 6.82, 95% CI: 3.94-11.81, *p *< 0.001). THD was inversely associated with mortality (OR 0.93, 95% CI: 0.88-0.98, *p* = 0.008), possibly reflecting early deaths in more severe cases.

Conclusions: In this Appalachian cohort of adult burn patients, mortality was significantly associated with larger burn size, prolonged ventilation, inhalation injury, smoking, and comorbidities such as diabetes, COPD, and home oxygen use. These findings highlight the need for individualized, multidisciplinary care strategies in resource-limited rural settings. Efforts to standardize inhalation injury diagnostics and enhance access to burn care may improve outcomes. Future studies should focus on scalable interventions and policy changes to reduce disparities in burn care and improve survival in underserved populations.

## Introduction

Despite recent medical innovations, burns remain a leading cause of morbidity and mortality worldwide. Appalachian areas are hit especially hard by this due to the unique challenges they face, such as decreased access to specialized care. The World Health Organization (WHO) estimated that 180,000 deaths every year are caused by burns, and non-fatal burns still cause serious injury, most commonly amputation [1,2]. This makes understanding the clinical characteristics and prognostic factors of burn patients integral in being able to give these patients the best recovery while also informing caregivers when cases contain a high risk of mortality.

There are many clinical factors that can contribute to the recovery of a patient. These include smoking history, age, severity of the burn, and the presence of inhalational injury. Comorbidities such as diabetes seem to play a prognostic role as well. Studies have shown that high blood glucose levels have been linked to an increased risk of complications, such as wound infections, bacteremia, and pneumonia [3]. Another study in 2015 linked diabetic burn patients to a longer hospital stay and a more intense need for surgery [4]. While there have been many studies on pediatric and elderly burn patient populations, this study investigates the mortality of patients aged 18-65 and their mortality rates after visiting the only Burn Intensive Care Unit (BICU) in West Virginia, Cabell Huntington Hospital.

West Virginia and the surrounding Appalachian region have long faced significant challenges related to limited resources, healthcare disparities, and inadequate access to appropriate medical facilities. The population is also disproportionately affected by chronic comorbidities. For instance, 15.0% of West Virginia adults have diabetes, compared to the national average of 11.6%, and 24.8% are smokers, exceeding the national rate of 19.8% [5,6,7,8]. Burns are shown to disproportionately affect low- and middle-income communities, which adds another layer of complexity to the Appalachian population [1]. While we have numerous comorbidities that predispose our community to worse burn injury outcomes, West Virginia is still left with one BICU in the entire state, having only six available beds. This is objectively problematic as we are left trying to decipher where to allocate this valuable resource.

This study aims to investigate the factors that influence mortality in burn patients aged 18 to 65. This age group has often been overlooked in previous research, as they typically do not present with the same complexities seen in pediatric or elderly populations. Gaining insight into mortality predictors in this demographic can not only guide clinicians in more effectively allocating resources but also provide a baseline for comparison, highlighting how much more vulnerable higher-risk populations may be when exposed to similar injuries. We will investigate this issue by identifying prognostic factors associated with mortality among burn patients at a BICU in rural Appalachia. Specifically, we investigate the roles of comorbid conditions, injury severity, inhalation injuries, and other clinical variables and how they influence patient outcomes. By analyzing these characteristics, we seek to provide actionable insights that can inform targeted medical interventions and induce policy changes to improve the delivery of care in rural settings.

## Materials and methods

The study was approved by the Marshall University Institutional Review Board #1 (approval no. 2063568-1; see Appendix for the approval letter). Patient records were retrospectively reviewed from the registry at Cabell Huntington Hospital, an academic teaching hospital, regional referral center, and verified trauma center in West Virginia, USA. The analyzed records belonged to patients admitted between January 2017 and January 2023. Data collection involved collaboration with the Information Technology (IT) department to extract relevant variables, including demographic, clinical, and burn-specific characteristics. This included age, gender, total hospital duration (THD), total ventilation duration (TVD), smoking history, diabetes mellitus (DM), home oxygen use, presence of chronic obstructive pulmonary disease (COPD), source of burn, inhalational injury, total body surface area burned (TBSA), and body mass index (BMI). 

Inhalational injury was diagnosed based on clinical observations, such as the presence of soot or carbonaceous material in the airway or difficulty in oxygenation. The total body surface area burned was calculated using the Wallace rule of nines. After reviewing the records, patients with non-burn diagnoses or insufficient documentation were excluded, leaving a final sample size of 748 patients. Data analyses were performed using Python and R software. Descriptive statistics summarized the patient characteristics. Categorical variables were expressed as counts (N) and percentages (%), while continuous variables were presented as means ± standard deviations (SDs). The chi-square test was used to compare categorical variables between survival groups (alive vs. dead). For continuous variables, Student’s t-tests or Mann-Whitney U tests were applied based on the distribution of the data. Logistic regression models were developed to assess the association between potential predictors and discharge status. Statistical significance was defined as a two-tailed p-value < 0.05.

## Results

Table 1 summarizes the demographic and clinical characteristics of the cohort, stratified by survival status. The majority of patients (552, 74%) were male. Gender distribution did not show statistically significant differences between survivors (N = 721) and deceased patients (N = 27). COPD was present in 14% of the patients, and there was a significant difference between the survivors and deceased patients (P ≤ 0.001). Home oxygen was present in 9.9% of the cohort. The survivors had home oxygen used in 8.2% vs. 55.6% of deceased patients (p < 0.001). Inhalation Injuries were present in 12% of survivors compared to 54% of deceased patients (p < 0.001). Cigarette smoking (p = 0.045) and home oxygen use (p = 0.031) were more prevalent among deceased patients with a significant difference between the two groups.

**Table 1:** Sample characteristics by survival status. Data presented as N (%). N, number; %, percentage; SD, standard deviation; χ2, chi-square test; COPD, chronic obstructive pulmonary disease; CIG, history of cigarette smoking; DM, diabetes mellitus. Statistical significance was defined as p-value <0.05.

Variables	Overall (N = 748)	Alive (N = 721)	Dead (N = 27)	p-value	χ²
Gender				0.85	0.04
Male	552 (73.8)	533 (73.9)	19 (70.4)		
Female	196 (26.2)	188 (26.1)	8 (29.6)		
COPD	106 (14.2)	89 (12.3)	17 (63.0)	<0.001	50.74
Home oxygen	74 (9.9)	59 (8.2)	15 (55.6)	<0.001	60.31
CIG	353 (47.2)	331 (45.9)	22 (81.5)	0.001	11.83
DM	104 (13.9)	90 (12.5)	14 (51.9)	<0.001	30.49
Inhalation injury	129 (17.2)	102 (14.1)	27 (66.7)	<0.001	70.56

Table 2 shows the demographic and clinical characteristics of the cohort, stratified by survival status. The mean age of survivors was 40 years (SD ± 14), compared to 57 years (SD ± 12) among deceased patients (p < 0.001). Survivors had a mean hospital stay of 9.2 days (SD ± 8), while deceased patients averaged 15.3 days (SD ± 10) (p = 0.012). Total ventilation duration was longer for deceased patients, with a mean of 18.5 days (SD ± 14), compared to 6.7 days (SD ± 9) for survivors (p < 0.001). Survivors had an average of 6% TBSA (SD ± 8), while deceased patients had a significantly higher mean of 28% (SD ± 20) (p < 0.001). 

**Table 2:** Sample characteristics by survival status. Data presented as number with SD; N, number; SD, standard deviation; THD, total hospital duration; TVD, total ventilation duration; BMI, body mass index; TBSA, total body surface area burn. Age presented in years. THD and TVD presented as days. TBSA presented as percentage. Statistical significance was defined as p-value <0.05.

Variables	Overall (N = 748)	Alive (N = 721)	Dead (N = 27)	p-value	t-score
Age	41.3 ± 13.8	40.2 ± 13.4	56.1 ± 12.8	<0.001	6.78
THD	9.5 ± 15.9	8.9 ± 14.8	15.3 ± 11.2	0.012	2.52
TVD	9.5 ± 14.5	8.7 ± 12.3	18.5 ± 14.1	<0.001	4.12
BMI	29.1 ± 9.5	28.8 ± 9.3	31.5 ± 11.2	0.062	1.86
TBSA	8.1 ± 11.1	6.3 ± 8.7	28.3 ± 15.6	<0.001	8.54

Table 3 presents the logistic regression model results. For TBSA, for each 1% increase, mortality risk increased by 1.15 times (95% CI: 1.10-1.21; p < 0.001). Prolonged ventilation duration was associated with a 1.08 times higher risk of mortality (95% CI: 1.02-1.14; p = 0.009). Patients with a history of smoking had 2.34 times higher odds of mortality (95% CI: 1.15-4.78; p = 0.018). These injuries were strongly predictive of mortality, increasing the odds by 6.82 times (95% CI: 3.94-11.81; p < 0.001).

**Table 3:** Association between predictors and survival status by using the logistic regression models. TBSA, total body surface area burned; TVD, total ventilation duration; THD, total hospital day. Statistical significance was defined as p-value <0.05.

Variables	Odds Ratio	95% CI	p-value
Age	1.06	1.00, 1.13	0.069
TBSA	1.15	1.10, 1.21	<0.001
TVD	1.08	1.02, 1.14	0.009
CIG	2.34	1.15, 4.78	0.018
Inhalation Injury	6.82	3.94, 11.81	<0.001
THD	0.93	0.88, 0.98	0.008

## Discussion

The focus of this study was to investigate the clinical characteristics and demographic factors of the group and how they correlated with the mortality risk in burn patients at the BICU at Cabell Huntington Hospital in the state of West Virginia. We investigated several known prognostic factors of burn mortality and highlighting smoking history and diabetes, which have been less explored in previous work. Out of the 748 patients in our study, 27 of them succumbed to their injuries, resulting in a 3.6% mortality rate. The American Burn Association (ABA) reported 29,165 national hospital burn admissions per year with an overall mortality of 2.7% [9]. Other studies not on the national scale have reported higher mortality rates, with another study of 18 BICUs encompassing 167 patients had 62 deaths - a 37.1% 90-day mortality rate [10]. Another international study concluded a 23.4% mortality rate [11]. The mortality rate seems to vary between 3% and 55% based on the clinical characteristics of the cohort, socioeconomic status of the treatment area, and severity of burns in the patients studied [12]. The mortality rate in our study was on the lower end of the range but still above the national average from the ABA. This shows a need for an increase in burn care quality and higher allocation of resources to the accessibility and understanding of the intricacies of burn care.

The average age of our population was 41.3. The median age of the population of West Virginia is 42.8 compared to the national median of 39.2 in 2023 [13]. This average age seems to be higher than other studies, with the Huang study having a median age of 38 years [10], and others reporting a mean age of 34.5 [14] and 24.07 in an international study [15]. This difference is due to the larger elderly population in West Virginia, which underscores the unique challenges our population faces when it comes to healthcare. The median age of surviving patients was 40 years, compared with the median age of 57 years for deceased patients (p < 0.001). Older patients are known to have much poorer outcomes than the healthier, younger population. Because of this, multiple burn mortality prediction models use age as a strong factor in determining patient mortality (e.g. Baux and modified Baux). A reason for this could be the immunologic response that may be lacking in the elderly patient. A 2016 study showed that elderly burn patients had higher mortality and Baux scores [16]. This indicates the importance of age in the prognosis. The study also suggested the reason for this was an immunocompromised state in the patient that did not allow them to respond adequately to the injury. This results in the lack of inflammatory response early, followed by a hyperinflammatory phase later in the recovery process [16]. This could pave the way for infectious complications such as sepsis, impaired wound healing, or a nosocomial infection while undergoing treatment. One meta-analysis in 2017 showed that most burn injuries in older patients occur in domestic settings and are associated with household tasks such as cooking. They too found hypertension and diabetes to be the most common comorbidities in their cohort [17], which are precisely the conditions that seem to affect our population in greater numbers. This creates the need for extra care to be provided to this unique group and a multidisciplinary approach when treating them, especially in our resource-limited area.

A unique finding in our study is that gender plays no statistically significant role in patient mortality. Other studies show that females have a higher mortality than males, even though males tend to make up more of the patient population. Mehta et al. used the 2023 WHO Global Burn Registry to show that in-hospital death was 2.16 times higher among females than males [18]. This difference could be attributed to the fact that our population of burn patients is predominantly male at 73.8%. This abundance of male patients could be due, in part, to the riskier occupational and recreational practices in this area. For example, trades are a large part of our economy, with a lot of people working in production factories or other potentially risky environments that could predispose them to burn injuries in these traditionally male-dominated occupations. Even though this may be a factor influencing our cohort, other studies have the same spread, with most burn patients being male, with the two previously mentioned studies having a 62% and 72.8% male population, respectively [18]. The ABA reports a nationwide admission rate of 66% male and 34% female [9]. Even at a national level, males are still more predisposed to burn injuries, however, our male population is over this average. This may bring some validity to the unique characteristics of our male burn population mentioned previously.

Total hospital duration (THD) proved to be a statistically significant prognostic factor of mortality, with survivors having a mean hospital stay of 9.2 days compared to the 15.3 days in the deceased population, resulting in an average THD of 9.5 days (p = 0.012). Another study reported a high variability in their cohort's THD, but a median of five days [19]. This study also correlated older patients to a longer THD and considered co-existing physical and mental conditions that the burn patient had and how that influenced their length of stay in the hospital. As THD increases, there is an increase in the risk of nosocomial infection, with *Staphylococcus aureus* being the most common [20]. This increased risk does not only explain a potential further increase in THD but also could lead to an increase in mortality, especially in elderly populations where immunocompromised states and decreased nutritional status are common. One study found an increase in THD of one week with each complication, with infectious complications being the most common, followed by cardiovascular and pulmonary complications [21]. These findings suggest a strong correlation between THD and mortality and can shed light on what to consider when caring for patients so providers can accurately inform patients and their families of the plan of care and prognosis.

Deceased patients also required more prolonged ventilation compared to survivors (18.5 days vs. 6.7 days, p < 0.001). Prolonged hospital stay can be linked to mortality due to the severity of the burns in these patients and other complications such as infections. The TVD findings can also be attributed to increased burn severity and the prevalence of complications that can be associated with prolonged ventilation. The ABA found that severe burns requiring surgery and mechanical ventilation carried a mortality rate of 17.8%, whereas burns requiring surgery and no ventilation had a 2.6% mortality rate [9]. This finding is also supported in the linear regression model, which showed that prolonged ventilation was associated with a 1.08 times higher risk of mortality. This increase in mortality could be due to the intrinsic severity of injury that is present in the patients who require ventilation. Regardless, ventilation should be reserved for the most appropriate circumstances, as mortality rates strongly increase once this is performed.

TBSA has historically been a crucial indicator of burn patient outcomes and has been the subject of many studies, with one concluding a cutoff burn size for mortality in adults to be 40%. At this TBSA, sepsis, organ failure, infections, and death were much more prevalent [22]. Burn mortality predictive scores such as the Baux, modified Baux, Abbreviated Burn Severity Index (ABSI), and Belgian Outcome in Burn Injury (BOBI) are widely used to predict prognosis and include TBSA as one of the factors influencing mortality. Our findings showed that survivors had a TBSA of 6% compared to a significantly higher mean of 28% in the deceased population (p < 0.001). This may reflect the increased inflammatory response to the more severe burn, as well as increasing the risk of infection and sepsis with increased TBSA. When patients have a higher percentage of their body burned, the body develops metabolic imbalances and a systemic inflammatory response. This, if severe enough, can lead to multi-organ failure and subsequent death. This is preceded by edema, shock, and cardiac, pulmonary, or renal dysfunction [23]. Higher TBSA is also associated with a delay in wound healing and an increased risk of systemic infection [24]. This finding is substantiated by the linear regression model, which showed a 1.15 times increased mortality risk with every 1% TBSA increase. TBSA should continue to be held near the top of medical decisions when allocating resources to burn patients.

Another previously known predictor of mortality is inhalation injury, which is estimated to affect up to one-third of the burn patients [25]. This has been proven so significant that the Baux score was modified to include the presence or absence of inhalational injury when determining prognosis. Our findings show an average inhalation injury of 17% in our population, with 12% surviving compared to the 53% of patients who succumbed to their injuries (p < 0.001). One study looked specifically at inhalational injury and thus had a higher percentage of affected patients (43.9%) and still had the same results as in our study [26]. This may also positively correlate to the prolonged TVD and THD findings previously discussed, as we showed that inhalation injuries were a strong predictor of mortality with almost a seven times increase. This is due to a combination of direct injury to the respiratory tract due to the heat and the injury from toxic gases such as carbon monoxide that dramatically increases fatality, especially when coupled with a high TBSA [26]. Inhalation injury remains complicated to measure as it is quantified differently depending on the treatment center. Since it is such a large predictor of burn patient mortality, there would be much benefit in the creation of standardized inhalation injury diagnostic criteria.

Other factors that significantly impacted patient mortality were cigarette smoking (p = 0.001), COPD (p < 0.001), and home oxygen use (p < 0.001), which were all found to be more prevalent in the deceased populations. This could be explained by a potential dramatic increase in mortality when cigarette smoking or COPD is combined with a sustained inhalation injury due to pre-existing damage to the respiratory system. Furthermore, 47.2% of our population were cigarette smokers compared to the national average of 11.6% in the US as of 2022, according to the Centers for Disease Control and Prevention (CDC) [6,7]. Cigarette smoking can not only delay wound healing and create cardiovascular issues but is also implicated in the development of acute respiratory distress syndrome (ARDS), with one study finding a twofold increase in the development of ARDS with tobacco use [27]. Interestingly, another study showed a decrease in mortality in burn patients who smoke (9% compared to 26%, p < 0.01), and they postulate a pro-inflammatory response in the smokers that give them an advantage when they suffer an acute inhalation injury due to a burn [28]. Nevertheless, smoking should be considered a risk factor for burn patient mortality due to the systemic injury it causes and the increased risk of complications shown in other work. This can be seen in the regression model that showed patients with a history of smoking had 2.34 times higher odds of mortality (p = 0.018). COPD and other chronic respiratory illnesses also increase the risk for ventilation and mortality [29]. Home oxygen use can carry its own risk for burn injury, with an estimated 2.98 per 1000 patients suffering a burn [30]. This is due to the ability of oxygen to accelerate the burning process of anything flammable that is nearby, such as a match or cigarette. While this is something that healthcare providers and patients need to be aware of, especially in our area where respiratory diseases are more common, home oxygen prescriptions should not be avoided since the benefits greatly outweigh the risk of burns. 

The source of the burn did not show statistical significance, which goes against previous studies where the etiology of the burn seemed to show statistical significance. A study in 2023 by Tasleem et al. found the etiology of the burn to be a statistically significant prognostic factor [31]. A potential explanation could be the population we serve in rural Appalachia, where there may be less of a variety of burn etiologies being seen in the BICU compared to a national scale. Another variable that was measured, while not statistically significant, was BMI. The average BMI of our population was 29.1. This places our cohort above the national average of BMI for men and women, which is 26.6 and 26.5, respectively [32]. While this was not a statistically significant predictor of mortality for our population, it should still be considered when delivering care due to the numerous co-morbidities that can accompany a high BMI.

The last statistically significant predictor of mortality in our patient cohort was diabetes mellitus (DM) status. Diabetes is known to complicate healing and increase the likelihood of infectious complications, along with a decreased rate of healing due to ischemic changes associated with diabetes. It can also be associated with more extreme burn severity due to potential neuropathy in the extremities, causing decreased sensation to the source of the burn. Previous studies have shown an increase of morbidity in patients with DM, but no increase in mortality [4]. The findings achieved in our study could be due, in part, to the higher diabetic population in our region. It could also be more dependent on other clinical factors previously mentioned.

The study has intrinsic limitations in that it investigates a single BICU in West Virginia, which could limit the applicability of the findings to the general population. However, it is important to understand the unique challenges and clinical characteristics that our rural, resource-limited population faces to optimize care and best predict mortality outcomes for our patients and caregivers. Another limitation in our study was the fact that the studied population came from a single burn unit. We attempted to ameliorate this by including many patients in the study over a long period of time.

## Conclusions

In this study, we highlight the key factors that can predict mortality rates in rural Appalachian burn patients between the ages of 18-65. These factors included age, THD, TVD, inhalation injury, and comorbidities such as DM, COPD, cigarette use, and home oxygen use. Knowing these prognostic factors allows us to tailor treatment regimens and customize medical care, which is especially important in the resource-limited and medically underserved rural Appalachia. To do this, there must be implementation of these findings throughout the BICU and the other disciplines that serve as a point of care to our unique population. A mentioned recommendation could be standardized inhalation injury diagnostic criteria to promptly address these injuries in our patients. Future resources and studies should aim to identify ways to equalize access to healthcare for rural Appalachian residents, produce novel treatment strategies for the described clinical factors, and enhance burn care availability and accessibility for all patients.

## Figures and Tables

**Figure 1:**
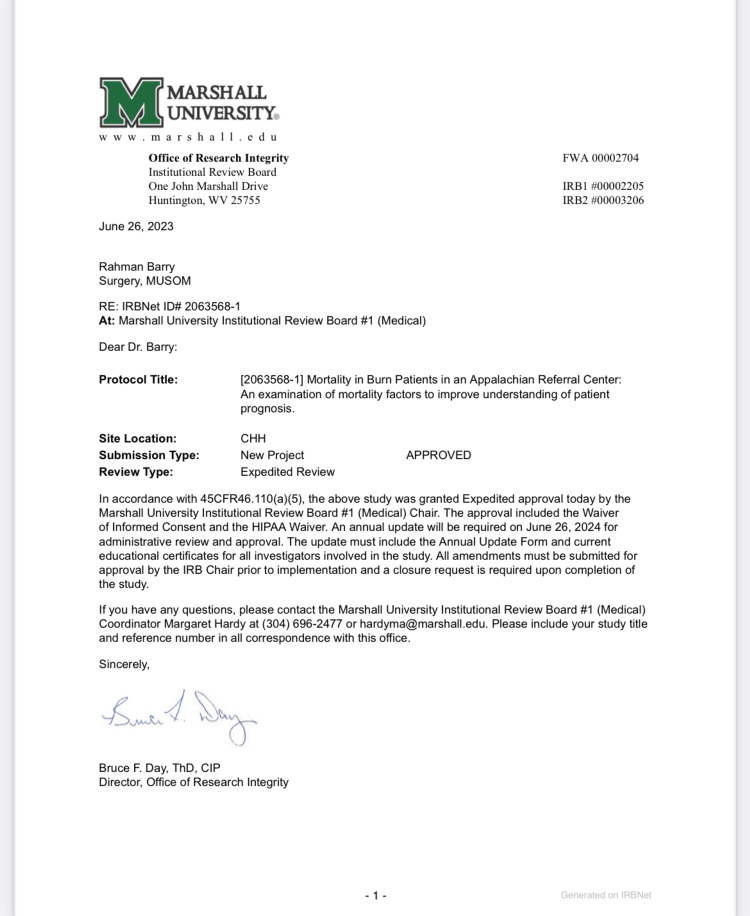
IRB approval letter
